# Crystallization Behavior and Properties of Glass Fiber Reinforced Polypropylene Composites

**DOI:** 10.3390/polym11071198

**Published:** 2019-07-17

**Authors:** Yuming Wang, Lihong Cheng, Xiaoqian Cui, Weihong Guo

**Affiliations:** 1 Shanghai Key Laboratory of Advanced Polymeric Materials, Key Laboratory for Ultrafine Materials of Ministry of Education, School of Materials Science and Engineering, East China University of Science and Technology, Shanghai 200237, China; 2 Department of Polymer Science, The University of Akron, Akron, OH 44325-3909, USA

**Keywords:** β-nucleating agent, glass fiber, polypropylene, crystallization, properties

## Abstract

Glass fiber with different content and different kinds of compatibilizers were used to prepare glass fiber-reinforced polypropylene (GFRP) composites. β-nucleating agent with different content was used to prepare β-polypropylene (PP), after which the toughness, crystallization ability and heat resistance were all enhanced. Differential scanning calorimetry (DSC) and wide-angle X-ray diffraction (WAXD) showed that the crystallite degree and crystallization ability were all greatly improved and β-PP was in dominant position. At last, both β-nucleating agent and glass fiber were used to modify the PP composites (β-GFRP). The formation of β-form PP made the matrix softer, which was beneficial for energy absorption and enhancement of toughness. The tensile strength, flexural strength and flexural modulus were improved dramatically, which were attributed to the coeffect of framework structure of GF and β-form PP.

## 1. Introduction

Glass-fiber-reinforced polypropylene composite material (GFRP) is a type of the material which has excellent mechanical properties, resistance to acid alkali, creep resistance, high thermal decomposition temperature and strong hydrophobicity [1,2,3,4]. Glass fiber (GF) is always served as reinforce material, to prepare GFRP, which is used in various economy and industrial fields such as electrical insulation materials, automobile dashboards, insulation materials and circuit board [1,2,3]. In order to obtain GFRP with high impact resistance, the common modification methods are grafting modification of polypropylene (PP) or adding elastomer modification. Grafting modification has no obvious effect on improving impact strength, while adding elasticity will reduce the rigidity [5,6]. Recently, many studies have focused on improving the interface compatibility of PP and GF, selection and rational use of compatibility agent [7,8]. There are relatively few studies on toughening by changing the crystal structure of the matrix PP itself, such as adding β-nucleating agent to induce the formation of toughening β-crystals [9,10]. As a semicrystalline polymer, polypropylene can form several crystal modifications, such as monoclinic α-form, trigonal β-form and orthorhombic γ-form [11,12,13]. The differences in super-molecular structures and crystalline states lead to different mechanical features. The α-form owns higher strength and modulus while the β-form yields better toughness but lower stiffness. The introduction of β-form crystal is one of the most effective methods to improve the toughness of PP [7,14,15,16]. Cui et al. have introduced N,N-dicyclohexyl-2,6-napthalene-dicarboxamide (TMB-5) into maleic anhydride-grafted polypropylene (PP-g-MAH) by extrusion blending, which lead to great increase of the mechanical properties [8]. Till now, four methods are used to gain β-PP [13]: (1) High supercooling degree [17]; (2) temperature gradient method [18,19]; (3) the shear [20,21] (4); adding β-crystal nucleating agent [12,22]. The first three methods are cumbersome, adding a β-nucleating agent is an easy and efficient way to improve the content of the β-PP in the process of PP commercial production [23,24].

In this study, the appropriate fiber glass content, the type of compatibilizer and the amount of addition were selected. Glass fiber-reinforced polypropylene (GFRP) composites were prepared by adding β-nucleating agent, PP-g-MAH or polyolefin elastomer grafted maleic anhydride (POE-g-MAH) to the optimized formulation. The crystallization behavior, microstructure of the composites, mechanical properties, thermal properties and melting behavior were investigated. The effect of the β-nucleating agent on the mechanical properties of the GFRP was studied in the paper.

## 2. Experimental

### 2.1. Materials

Polypropylene M1600 (melt flow rate: 25 g/10 min at 230 °C and 2.16 kg) was purchased from South Korea LG Chem (Seoul, South Korea). Glass fiber, ECS305K-3, was produced by Chongqing International Composite Material Co., LTD (Chongqing, China), short fiber with the length 3–4.5 mm and diameter of 13.1 µm. β-nucleating agent with the trademark WBG-II was a rare earth organic compound supplied by Guangdong Winner Functional Materials Co., LTD. (Yunnan, China). Maleic anhydride-grafted polypropylene (PP-g-MAH), CMG9801-GS, was produced by Nantong Sunny New Technology Development Co., Ltd. (Nantong, China). Small amount of antioxidant (Irganox 1010 and 168, commercially available) was also used to prevent the thermal decomposition during processing.

### 2.2. Sample Preparation

To minimize the effects of moisture, the used pellets were dried for 8 h at 80 °C. Melt blending of the composite was performed by a corotating twin-screw extruder (TSJ-20B, L/D = 42; Nanjing Tengda Equipment Limited Company, China) with the rotation speed of 120 rpm. The temperature along the barrel was from 130 to 190 °C. Blends were cooled down with water bath before they were pelletized. The obtained blends were dried at 80 °C in a drying oven. Then the pellets were injection-molded (CJ80M2, Chengde Plastic Machinery Limited Company, Chengde, China) into standard specimens for testing at the temperature from 200 to 230 °C, the mold temperature was 40 °C.

For compatibilizers-modified GFRP, two compatibilizers of different content (5, 10, 15 wt %) were added to the formula, and the antioxidant 1010 and 168 were 0.05% each. The PP-g-MAH-modified GFRP composites were named as GFRP-PP-5, GFRP-PP-10, GFRP-PP-15, and POE-g-MAH-modified GFRP composites were named as GFRP-POE-5, GFRP-POE-10, GFRP-POE-15.

For β-crystal-modified GFRP, the content of glass fiber was 30 wt %, the addition amount of β-nucleating agent was 0.05, 0.10, 0.15, 0.20 and 0.25%, respectively, which were labeled as β-GFRP-5, β-GFRP-10, β-GFRP-15, β-GFRP-20 and β-GFRP-25, respectively. In order to compare the effects of β-nucleating agent on the mechanical properties of PP, pure PP and GFRP samples were prepared under the same processing conditions, glass fiber content was 30 wt %, and the content of nucleating agent was 0 wt %.

### 2.3. Characterization

#### 2.3.1. Differential Scanning Calorimetry (DSC)

Samples were studied by DSC 200PC (NETZSCH) in a nitrogen atmosphere for the crystallization behavior and melting characteristics. About 10 mg samples were heated from room temperature to 200 °C at the rate of 10 °C/min, and held for 5 min to eliminate the thermal history. After that, the samples were cooled to 50 °C at 10 °C/min and then reheated to 200 °C with the heating rate of 10 °C/min. The degree of crystallinity (*X_c_*) was calculated from the data obtained during the second run by

(1) $$X_{c}=\frac{\Delta H_{m}}{\phi \Delta H_{m}^{o}}\times 100\%$$

where ϕ is the weight fraction of PP corresponding, Δ*H_m_* is the melting enthalpy value measured by DSC, and Δ*H_m_^o^* is the melting enthalpy of the completely crystalline PP, where the values of Δ*H_m_^o^* for α-PP and β-PP were 177.0 and 168.5 J/g, respectively [13].

The relative content of the β-phase PP (Φ_β_) was calculated according to the following relationship:

(2) $$\Phi_{\beta }=\frac{X_{\beta }}{X_{\beta }+X_{\alpha }}\times 100\%$$

where *X*_β_ and *X*_α_ are degree of crystallinity of β-PP and α-PP, respectively.

#### 2.3.2. Scanning Electron Microscope (SEM)

The specimens were cryo-fractured in liquid nitrogen and then the surfaces were investigated with a Hitachi S-3400 scanning electron microscope (SEM). To achieve good electric conductivity, all samples were coated with gold–palladium by a sputter coating instrument.

#### 2.3.3. Mechanical Properties

The tensile strength was measured using a computerized universal testing machine (WSN-20KN, Shenzhen) at a tensile speed of 50 mm/min according to ISO 527-2-1994. Notched Izod impact strength was measured by an impact tester (JJ-20, Changchun Intelligent Instrument and Equipment Limited Company) according to ISO 180-2001. The bending performance was tested according to GB/T9341-2008 and the pressing rate was set to 10 mm/min. The thermal deformation temperature was tested according to GB/T1634-2004. Measurements were carried out at room temperature, and 15 samples were tested from each composite and the average results were recorded.

#### 2.3.4. Wide-Angle X-Ray Diffraction (WAXD)

Wide-angle X-ray diffraction was obtained with a D-max2550 VB-PC X-ray Diffractometer. The scanning range was 5° to 50° with the rate of 3°/min and a step length of 0.02°. The Cu K-alpha irradiation source was operated at 40 KV and 100 mA. The K value representing the β-form content in PP was determined from X-ray diffractograms according to Turner-Jones et al. [14].

(3) $$K_{\beta }=\frac{H_{\beta (300)}}{H_{\alpha (110)}+H_{\alpha (040)}+H_{\alpha (130)}+H_{\beta (300)}}$$

where *H*_α(110)_, *H*_α(040)_ and *H*_α(130)_ are intensities of α-diffraction peaks corresponding to 2θ angles 14.2°, 17.0° and 18.8°, respectively, and *H*_β(300)_ is the intensity of β-diffraction peak at 2θ angle 16.2°.

## 3. Results and Discussion

### 3.1. Compatibilizer Modified Polypropylene

#### 3.1.1. Effects of Glass Fiber Contents on the Properties of GFRP Composites

**Figure 1:**
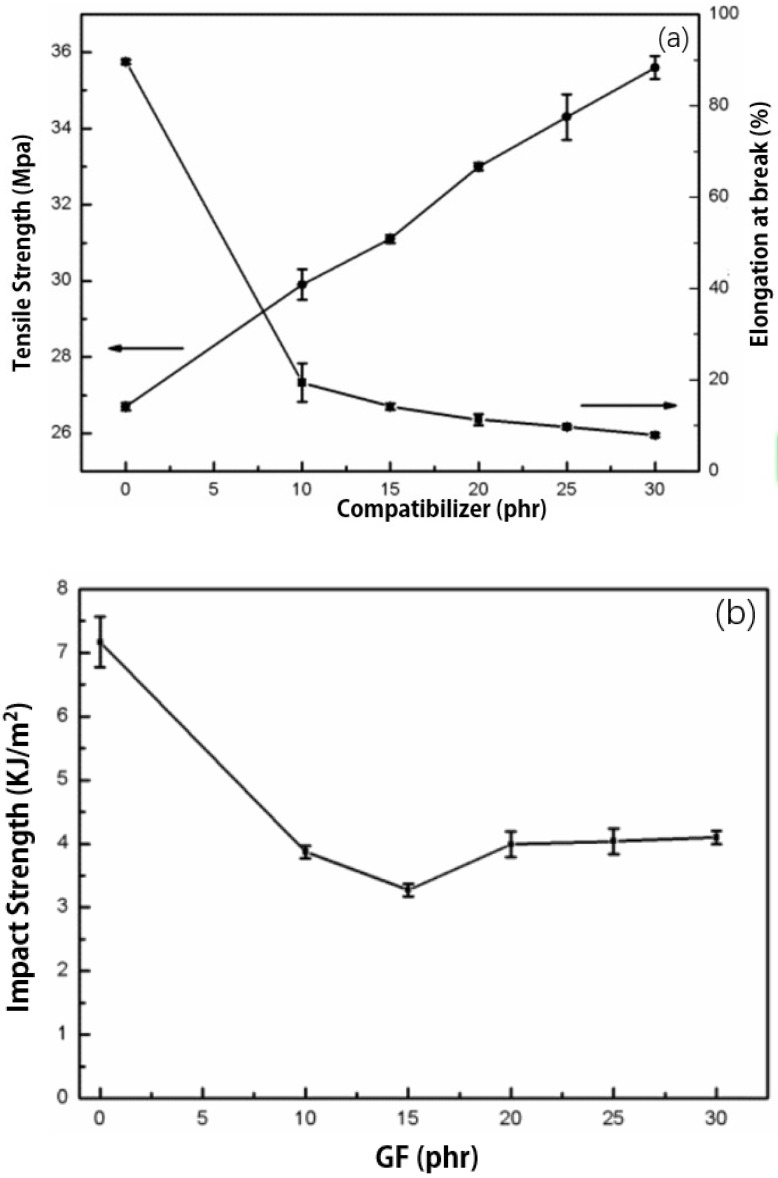
Effects of glass fiber contents on the properties of glass fiber-reinforced polypropylene (GFRP): (**a**) Tensile and elongation properties; (**b**) impact strength.

Figure 1 shows the relationship between the glass fiber content and the properties of GFRP composites. It can be seen from Figure 1a that the tensile strength of pure PP is 26.7 MPa. After the addition of GF, the tensile strength of the sample increases significantly, and it almost shows a linear growth with the increase of glass fiber content. The tensile strength of GFRP-30 reached a maximum value of 35.6 MPa. This is mainly due to the high strength of GF which plays a role as an enhancer in the material. When subjected to external stretching, GF can bear the external force, until the GF fracture, debonding or pull out. When GF content increased to a certain level to interlaced, a three-dimensional network structure was formed. When stretching, the stress received by the material is transferred from the matrix to GF, and GF is the main stress-bearing object, thus, the tensile strength increases with the increase of GF content. As GF is a rigid material, its shape variable is very limited, therefore, the elongation at the break of GFRP composite material is very low. Figure 1b shows that the impact strength of pure PP sample is 7.2 KJ/m^2^. After the addition of GF, the impact strength of GFRP sample shows a change trend of decreasing first and then increasing. When the GF content is low, GF does not play an enhancement role in GFRP composite, the impact strength of the material decreases. When GF intertwines with each other in GFRP, GF plays a role of skeleton, and the impact strength was improved.

#### 3.1.2. Crystallization and Melting Behavior of GFRP Composites with Different Glass Fiber Contents

**Figure 2:**
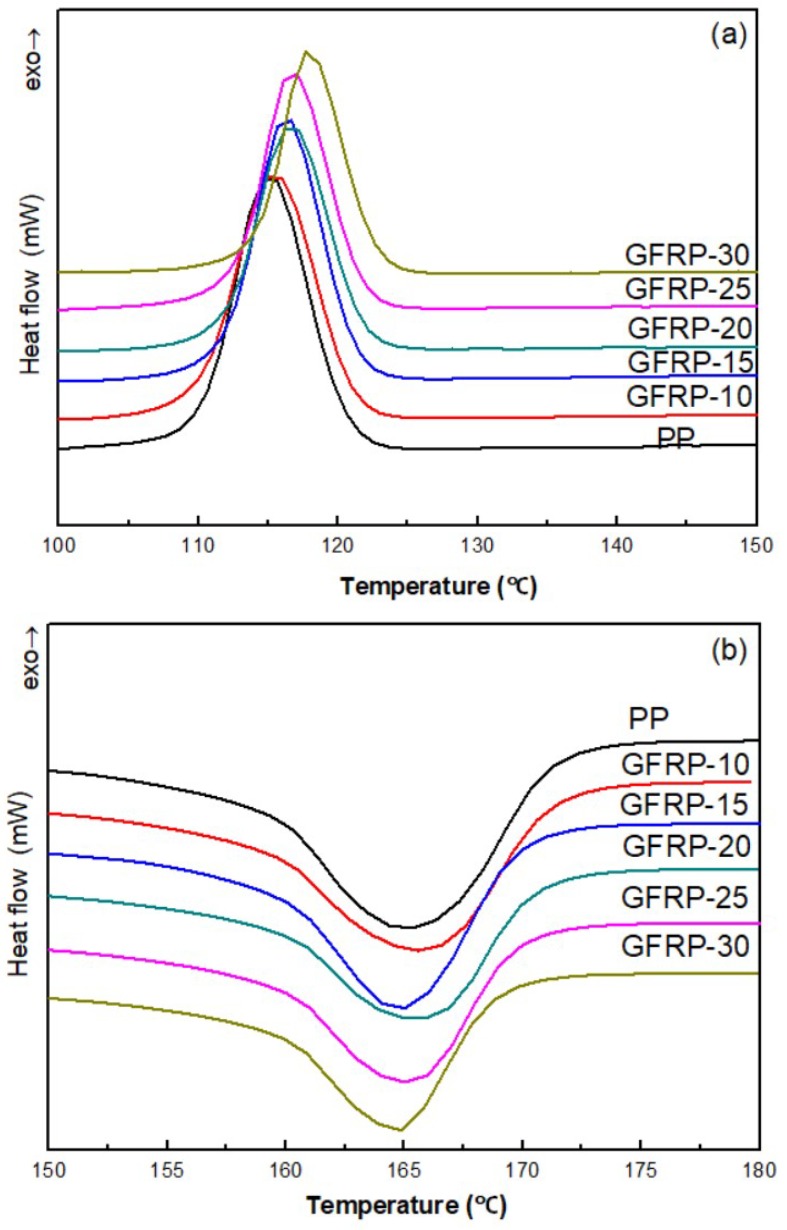
Effects of GF contents on the melting behavior and crystallization behavior of GFRP. (**a**) Differential scanning calorimetry (DSC) curves of the crystallization behavior; (**b**) DSC curves of the melting behavior.

Figure 2 and Table 1 are the DSC curves of the melting behavior and crystallization behavior of GFRP composites, respectively. It can be seen from Table 1 that the crystallization temperature of GFRP composite material moves towards the high temperature with the increase of GF content, but the variation of crystallization temperature is not significant. The melting temperature of GFRP composite is almost constant, with only one melting peak. The crystallinity of GFRP composites shows a trend of first increasing and then decreasing, since a small amount of GF could act as nucleating agent in the system, promoting the heterogeneous nucleation of PP and causing the improvement of crystallinity. However, when the amount of GF was larger, the presence of GF prevented the α-spherulites of PP from expanding in all direction, thus resulting in a decrease in crystallinity. Figure 2 also shows that GF has a nuclear capability. A small amount of GF can provide the nucleation center to induce the crystallization at high temperature, reduce the supercooling degree of the sample, speed up the crystallization rate, short the production cycle and greatly save the processing cost.

#### 3.1.3. Effects of Different Kinds and Contents of Compatibilizers on the Properties of GFRP Composites

**Figure 3:**
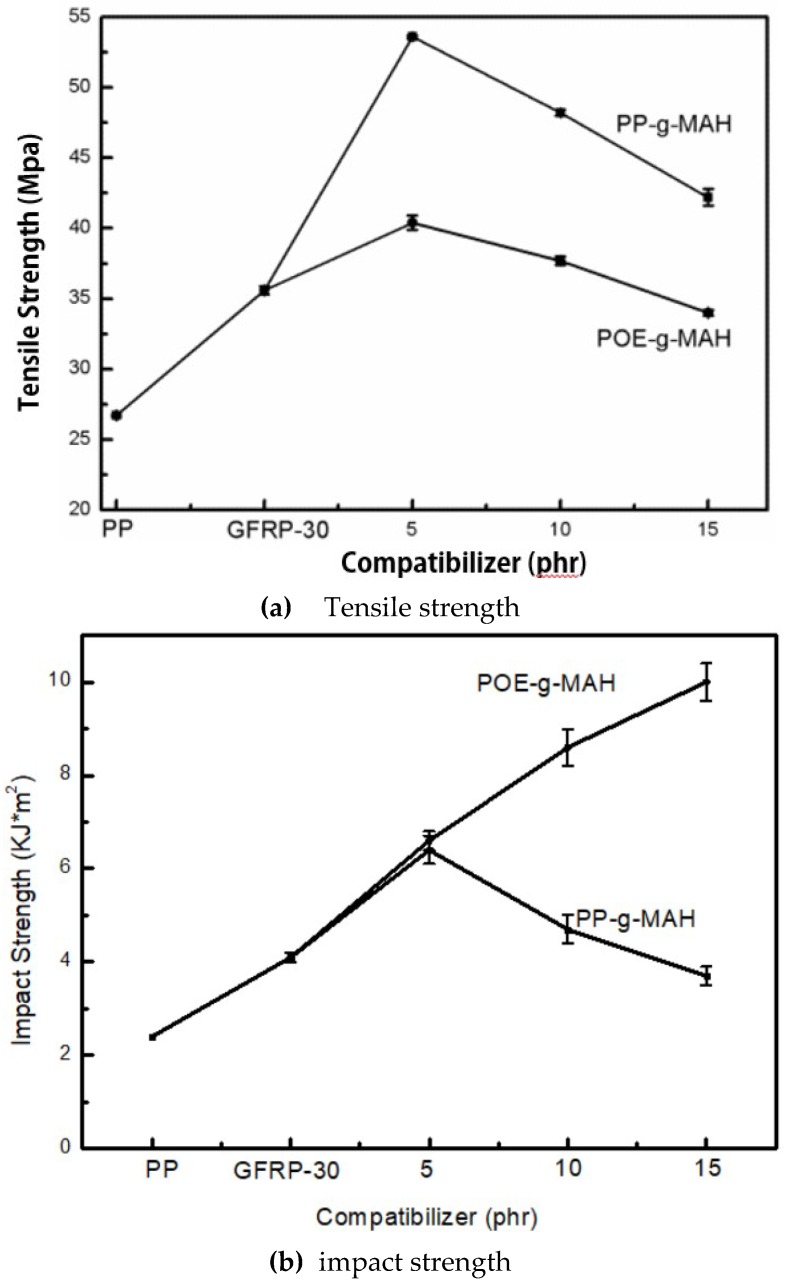
Effects of compatibilizers on the mechanical properties of GFRP (30 wt % GF): (**a**) Tensile strength; (**b**) impact strength. PP-g-MAH: Maleic anhydride-grafted polypropylene; POE-g-MAH.

GFRP composites with two compatibilizers (PP-g-MAH and POE-g-MAH) were prepared by selecting the formulation with the best comprehensive properties, namely GFRP-30, and their comprehensive mechanical properties were compared. Figure 3 is the effects of compatibilizers on the mechanical properties of GFRP (30% weight content of GF).

In Figure 3a, the tensile strength of the specimen with PP-g-MAH increases with the increase of the content of PP-g-MAH and then reduces. When PP-g-MAH content is 5 wt %, the tensile strength reaches a maximum 53.6 MPa which is 50.7% higher than GFRP-30 (35.6 MPa); 100.7% higher than PP (26.7 MPa). An appropriate amount of PP-g-MAH can effectively enhance the interface adhesion between GF and PP, making the fiber more difficult to break, pull out and disbond. For the samples with POE-g-MAH added, the tensile strength of the samples also showed a trend of first increase and then decrease with the increase of POE-g-MAH content. When the POE-g-MAH content was 5 phr, the maximum value was 40.4 MPa which is 13.4% higher than that of GFRP-30 (35.6 mpa) and 51.3% higher than that of pure PP (26.7 mpa). The tensile strength of GFRP-PP was significantly higher than that of GFRP-POE, which was mainly due to the physical compatibility between PP-g-MAH and matrix PP, while the compatibility between POE-g-MAH and matrix PP was poor. As the content of compatibilizer exceeded a certain value, GF were entangled with each other, not easy to disperse, and failed to give full play to the skeletal role of GF. Moreover, POE-g-MAH is an elastic particle, and the increase of elastic particles in the system will also reduce the tensile strength of the sample.

For PP-g-MAH modified GFRP-30 in Figure 3b, the impact strength of GFRP-30 composite material was 4.1 KJ/m^2^. The impact strength first increased and then decreased with the addition of PP-g-MAH. When the weight content of PP-g-MAH was 5%, the impact strength was the highest at 6.4 KJ/m^2^, which is 81.8% higher than that of GFRP-30. When the amount of PP-g-MAH was relatively small, one end of PP-g-MAH was physically compatible with the base PP, and the other end reacted with the coupling agent on the surface of GF with maleic anhydride, forming a more solid chemical bond and enhancing the interface compatibility between GF and the base PP. When more PP-g-MAH was added, the impact strength decreased. For the samples with POE-g-MAH added, the impact strength increased with the increase of the amount of POE-g-MAH, reaching a maximum of 10.0 KJ/m^2^, which was 144% higher than that of GFRP-30. POE can absorb a lot of energy and prevent the development of crazing; therefore, the impact strength was greatly improved.

#### 3.1.4. Crystallization and Melting Behavior of Compatibilizer Modified GFRP

**Figure 4:**
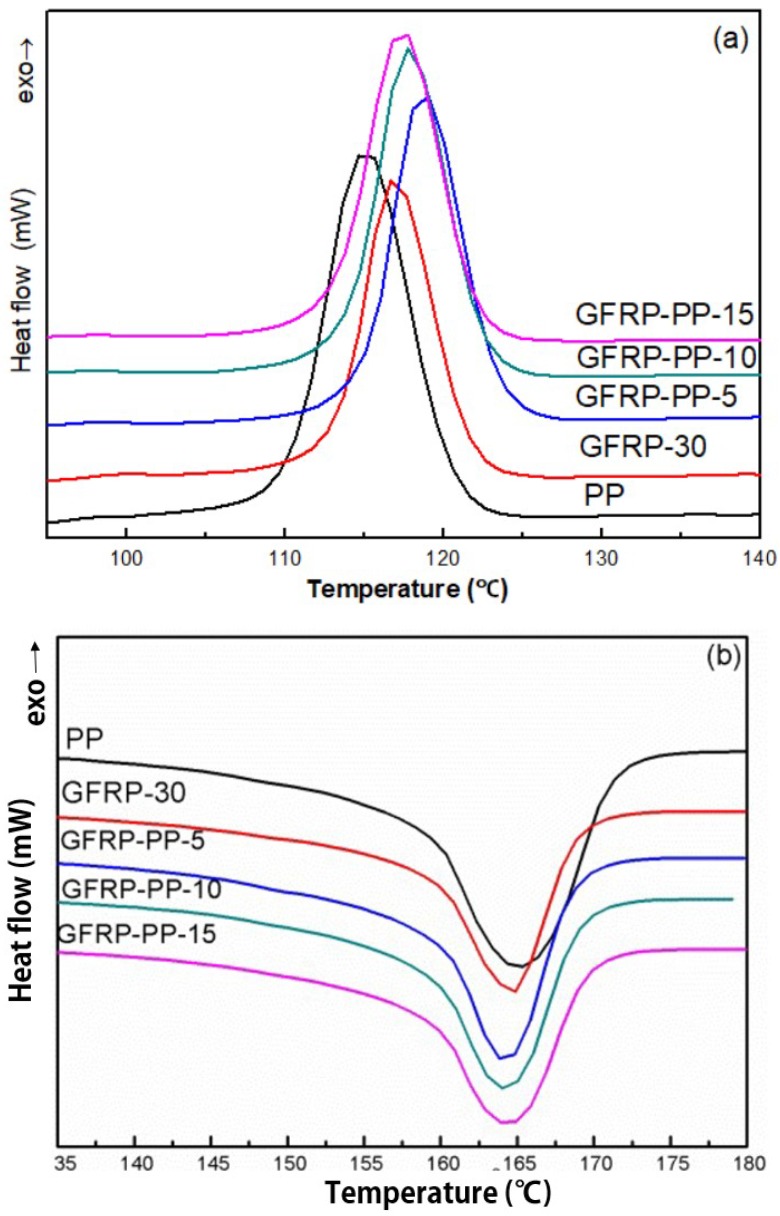
Effects of different ratio of PP-g-MAH on the crystal behavior and melting behavior of GFRP: (**a**) DSC curves of crystallization behavior; (**b**) DSC curves of melting behavior.

Figure 4 and Table 2 show the effects of different ratio of PP-g-MAH on the crystal behavior and melting behavior of GFRP. For PP-g-MAH-modified GFRP, the crystallization temperature was moving in the direction of high temperature and up to 118.7 °C while the crystallization temperature of pure PP was 115.0 °C and that of GFRP-30 was 116.9 °C. Adding compatibilizer cannot affect the melting temperature too much, but the crystallinities of samples were increased, this was due to the improved compatibility between GF and PP matrix. Glass fiber also provides nucleation surface for PP, which can promote the reduction of crystallization barrier of PP. PP first nucleated and crystallized on the surface of glass fiber, while the matrix far from GF has not nucleated due to the high potential barrier. As the crystallization temperature continued to decrease, the surface crystal of GF continued to grow, and the matrix far away from GF began to nucleate and crystallize when the temperature decreased to a certain level. Therefore, the crystallinity was improved to a certain extent compared with that of GFRP-30. Since the crystallization of the polymer is a regular and orderly arrangement of molecular chains to form a three-dimensional remote ordered crystal structure, the crystal type of PP is a large α-form, the addition of GF destroys this ordered structure and the crystallinity of the sample decreased compared with pure PP.

**Figure 5:**
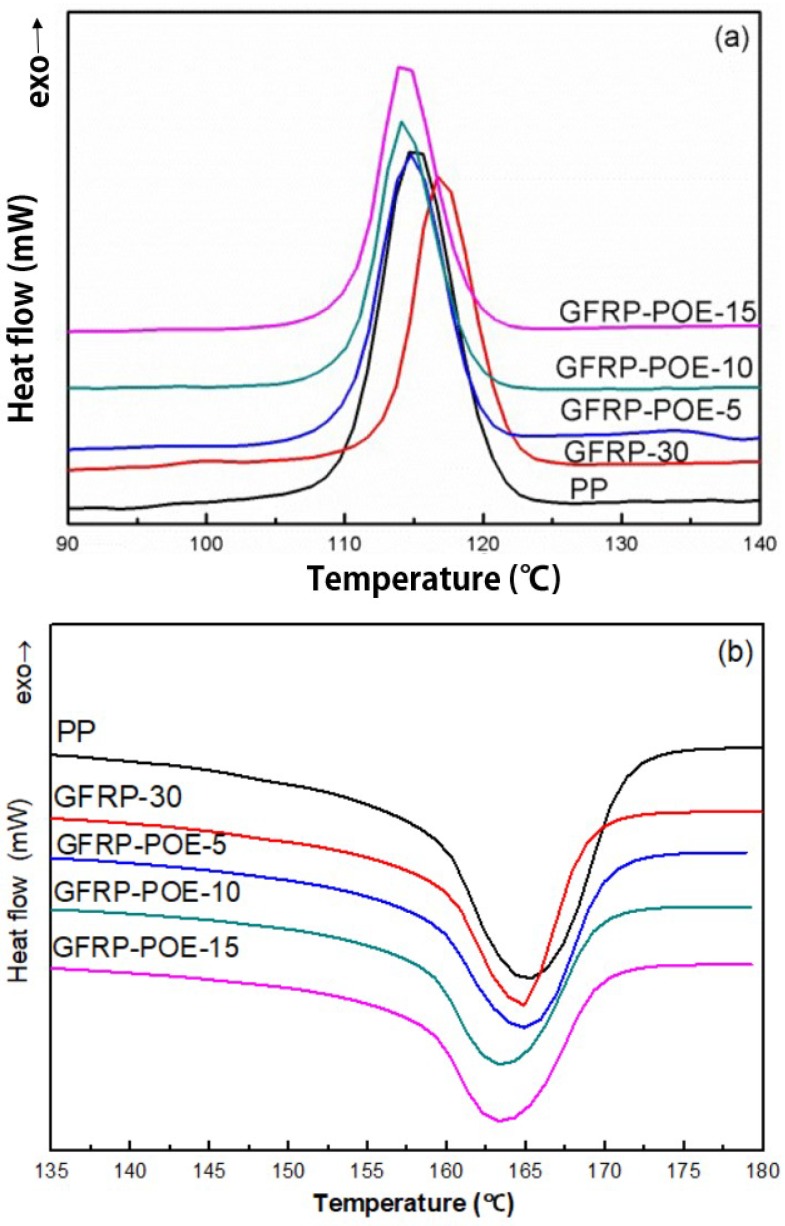
Effects of POE-g-MAH on the crystal behavior and melting behavior of GFRP: (**a**) DSC curves of crystallization behavior; (**b**) DSC curves of melting behavior.

Figure 5 and Table 2 show the effects of different ratio of POE-g-MAH on the crystal behavior and melting behavior of GFRP. In Figure 5, with the increase of the content of the compatibilizer POE-g-MAH, the crystallization temperature and melting temperature of the sample decreased slightly, and the crystallinity also decreased to a certain extent. POE-g-MAH had a limited effect on enhancing two-phase interfacial properties, and its compatibility with the matrix PP was limited. The melting temperature and crystallization temperature of POE-g-MAH were lower than those of PP, and the addition of POE-g-MAH can reduce the crystallization and melting temperature of samples.

#### 3.1.5. Microstructure of Compatibilizer Modified GFRP Composites

**Figure 6:**
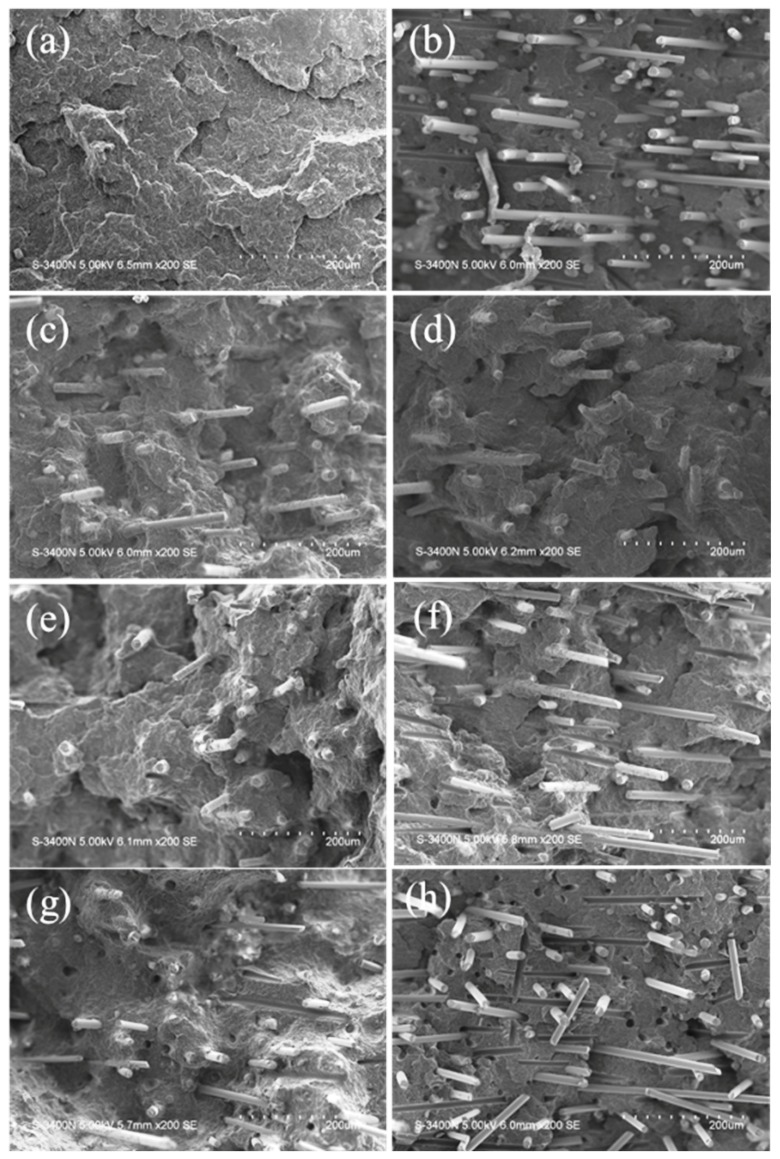
Scanning electron microscope (SEM) images of GFRP composites with different compatibilizers: (**a**) Pure PP; (**b**) GFRP-30; (**c**–**e**) GFRP-PP-5, GFRP-PP-10, GFRP-PP-15; (**f**–**h**) GFRP-POE-5, GFRP-POE-10, GFRP-POE-15.

Figure 6 is the SEM images of different kinds and contents of compatibilizer of GFRP composite. It can be seen from Figure 6a that for pure PP, the cross section is smooth and flat, which is a typical brittle fracture. The fracture cracks were coarse and few, and the shock energy was absorbed mainly through the induction and expansion of the craze. For GFRP-30, Figure 6b shows a certain degree of orientation of GF in the flow direction of matrix PP, and a large number of GF were pulled out to form holes. The surface of GF was smooth, indicating poor compatibility between GF and PP. In Figure 6c–e, surface folds on the cross section of the matrix increased after the addition of the compatibilizer PP-g-MAH, indicating that more energy was absorbed. GF had a certain orientation in the flow direction of PP, and the surface of GF adhered to polymers. PP-g-MAH was also a separate phase aggregated as a stress concentration point. As can be seen from Figure 6f–h, when POE-g-MAH was added, a large number of fibers were pulled out to form a large number of holes. As the POE-g-MAH content increased, the surface of GF was coated with fewer and fewer polymers. When the content of GF was 15 wt %, the surface of GF became very smooth; Figure 6f–h show that POE-g-MAH existed as a separate phase, which also explains the poor comprehensive mechanical properties of POE-g-MAH samples.

### 3.2. β-Crystal-Modified GFRP

#### 3.2.1. Crystallization Behavior and Melting Behavior of β-GFRP

**Figure 7:**
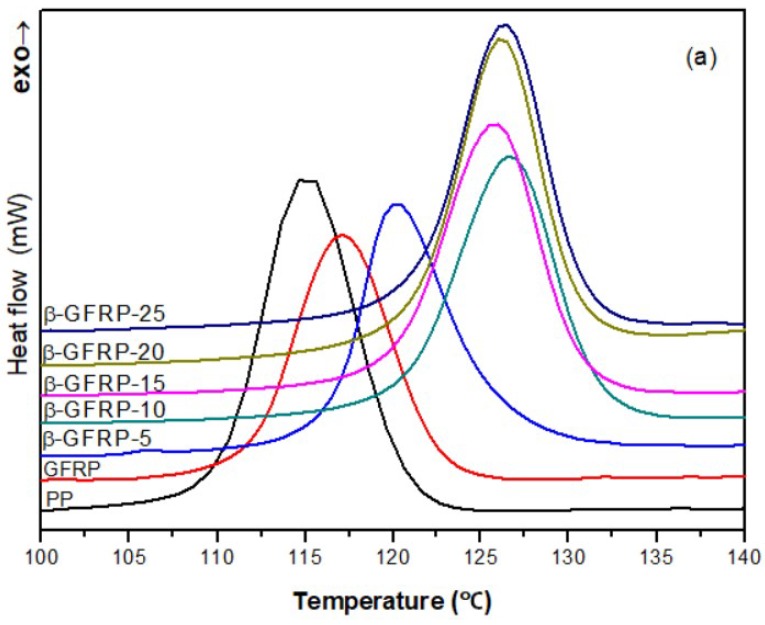

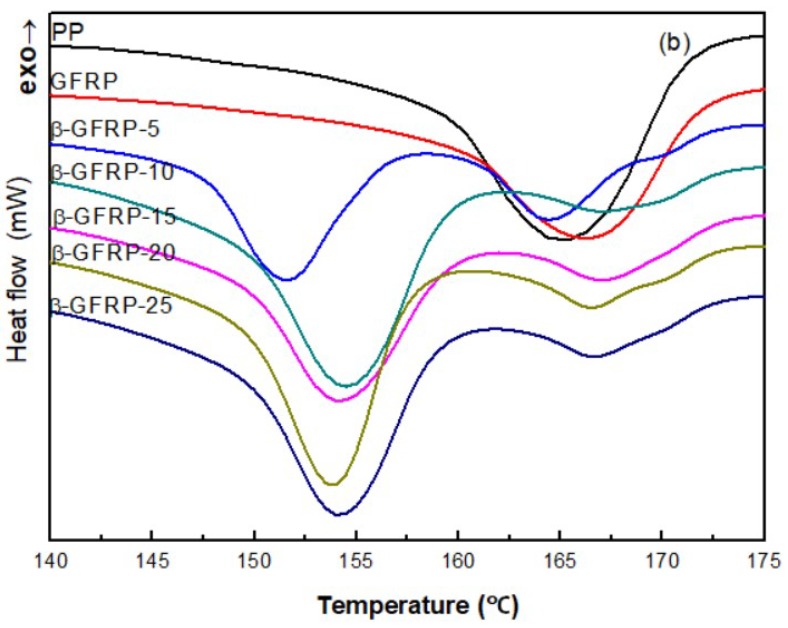
The crystal behavior and melting behavior of β-GFRP composites: (**a**) DSC curves of crystallization behavior; (**b**) DSC curves of melting behavior.

Figure 7 is the DSC crystallization curves of different β-GFRP composites. The corresponding data obtained from DSC analysis are listed in Table 3. Figure 7a shows crystallization temperature (T_c_) for pure PP is 115.0 °C. After adding glass fiber, the T_c_ increased to 117.2 °C. When the β-nucleating agent content reaches 0.10%, the T_c_ has improved significantly to about 126 °C. Figure 7b shows the DSC melting curves of different samples of β-GFRP composites. The melting curves of pure PP and β-GFRP show some differences. Pure PP shows single melting peak in the 164.9 °C. After adding glass fiber, the melting curve of the sample did not change too much, indicating that no β-crystal phase was formed. For β-GFRP, the melting curve changes obviously and shows double melting peaks. This is due to the successful formation of β-crystal phase by the addition of a β-nucleating agent. Since the melting point of β-crystal phase is lower than that of α-crystal phase, the low temperature peak on the left side of Figure 7b is the characteristic melting peak of β-crystal, while the high temperature peak on the right side is the characteristic melting peak of α-crystal. From the data in Table 3, with the increase of the content of β-nucleating agent, the melting temperature also slightly increased, and the relative area of the melting peak of β-crystal gradually increased. With the increase of the content of β-nucleating agent, the calculated relative content of β-crystal (ϕ_β_) increased significantly to maximum 92.8% when β-nucleating agent content was 0.10 wt %. This shows the crystal was mostly formed by β-crystal.

**Figure 8:**
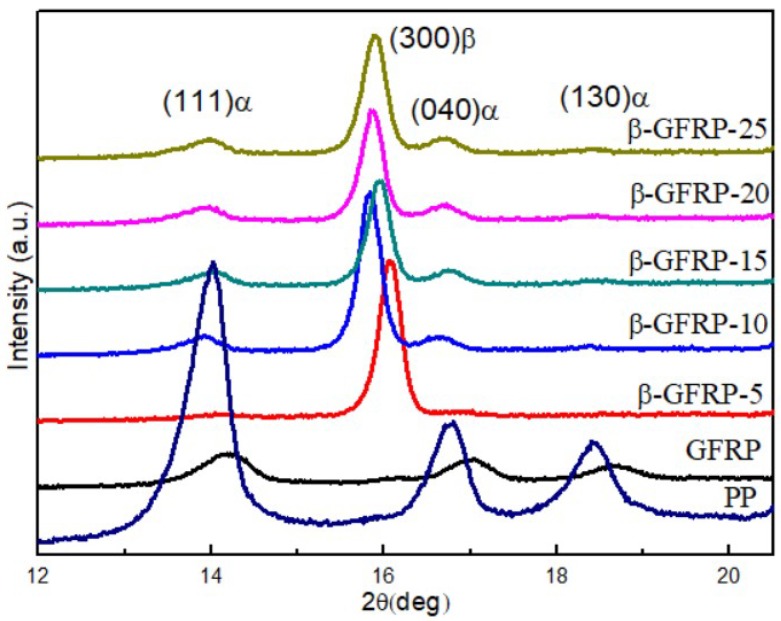
X-ray diffraction (XRD) patterns of β-GFRP composites.

Figure 8 is the WAXD test spectrum of β-GFRP composites with 0.10 wt % content of β-nucleating agent. There are three diffraction peaks of pure PP, respectively in 2θ = 14.2°, 16.8° and 18.6°, corresponding to α-crystal (111), (040), (130), respectively. Three diffraction peaks of α-crystal also appeared in the GFRP, but the intensity of diffraction peaks was obviously smaller than that of PP, indicating that GF has a destructive effect on PP crystallization. For β-GFRP specimens, the emergence of a new strong diffraction peak near 2θ = 16.0° corresponding to the β-crystal (300). This shows that the β-nucleating agent for PP has good induced crystallization. It can effectively reduce the crystallization of PP barrier and promote the crystallization of PP.

At the same time, the intensity of the three diffraction peaks corresponding to the α-crystal decrease significantly, indicating that most crystals in the sample are β-crystals. With the increase of the content of β-nucleating agent, the relative content of β-crystal tends to be stable. According to Equation (3), the relative contents of the crystals are calculated, and the data are summarized in Table 3. It is found that although the testing principles of DSC and WAXD are different, the trend of testing results of β-crystal is almost the same.

#### 3.2.2. Mechanical Properties of the Composites of β-GFRP

**Figure 9:**
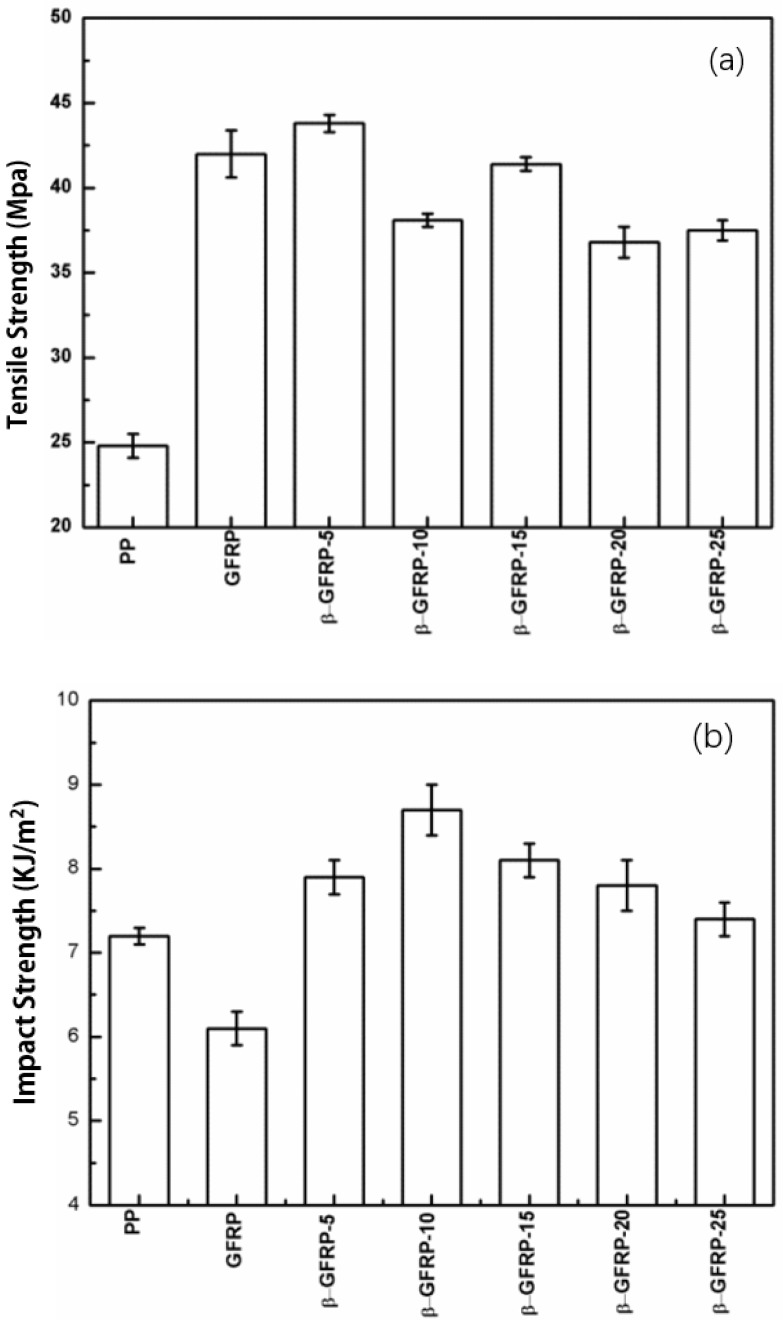
Mechanical properties of β-GFRP: (**a**) Tensile strength; (**b**) impact strength.

Figure 9 shows the mechanical properties of β-GFRP. In Figure 9a, the tensile strength of GFRP increased to 42.0 MPa with the addition of GF, which increased by 69.4% than 24.8 Mpa of pure PP. When the content of nucleating agent was 0.05%, the tensile strength of the sample was 43.0 MPa, 1.77 times as much as that of pure PP, which was mainly due to the joint action of β-crystal and GF skeleton. When more β-nucleating agent was added, the increasing degree of the tensile strength was reduced. This was due to the β-nucleating agent exceeding saturation concentration. Part of the β-nucleating agent did not dissolve in the PP phase but existed in the form of filler in PP, which made a lot of interface defects exist in the matrix. These defects act as the stress concentration points, which reduce the tensile strength.

In Figure 9b, the impact strength of pure PP is 7.2 KJ/m^2^ and the impact strength of GFRP specimens decreases obviously. The highest impact strength of samples with β-nucleating agent was 8.7 KJ/m^2^, as the β-nucleating agent content was 0.10 wt %, which is 20.8% higher than that of pure PP and 42.6% higher than that of GFRP, suggesting that the increase of impact strength was due to the addition of β-nucleating agent. The toughness of the composite was greatly enhanced by the smaller size of the β-crystal induced by β-nucleating agent. The layered structure of the β-crystal buffered the impact stress.

When the content of β-nucleating agent exceeded 0.10%, the content of β-nucleating agent was in a state of oversaturation. Part of the nucleating agent cannot be dissolved in PP and remained in its original form. At this time, the extra existed in the matrix PP in the form of filler, which is not conducive to improving the impact toughness as a defect or stress concentration point. The change of impact strength was consistent with the change of relative content of β-crystal obtained by DSC.

#### 3.2.3. Microstructure of β-GFRP Composite

**Figure 10:**
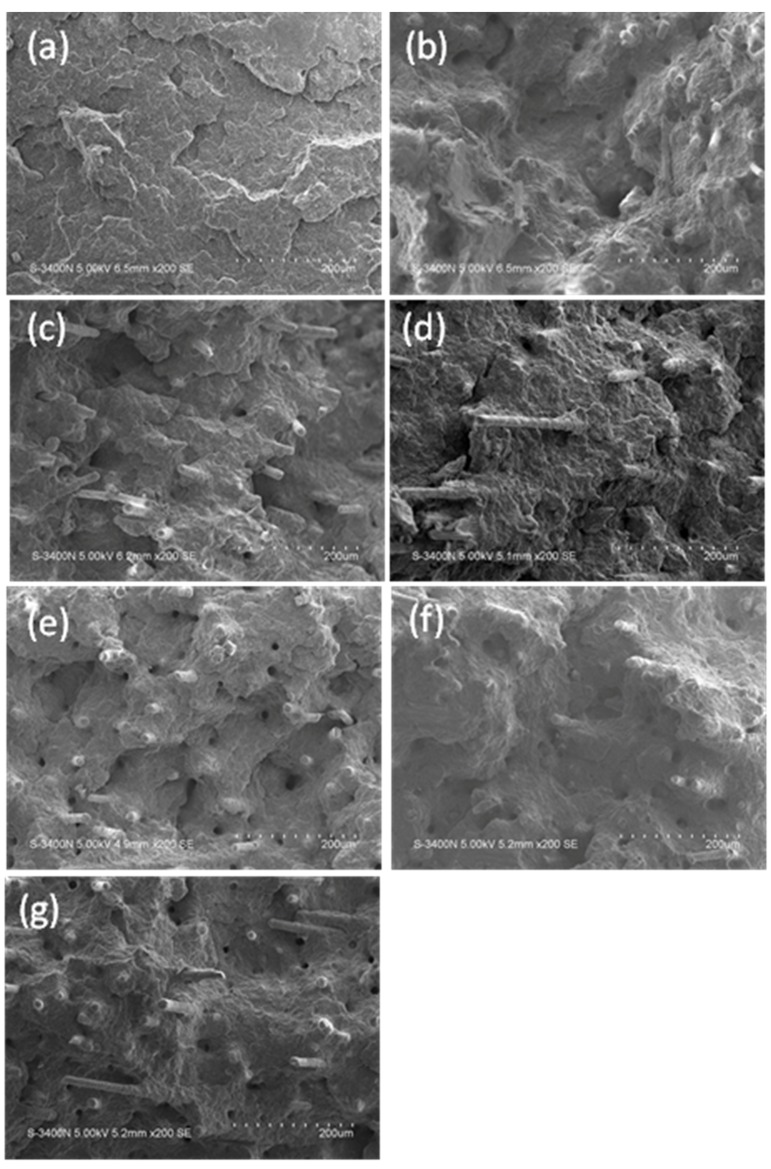
SEM images of PP, GFRP and β-GFRP. (**a**) Pure PP; (**b**) GFRP; (**c**) β-GFRP-5; (**d**) β-GFRP-10; (**e**) β-GFRP-15; (**f**) β-GFRP-20; (**g**) β-GFRP-25.

Figure 10 shows the SEM images of notched impact sections of different samples. As can be seen from Figure 10a, fracture surface for pure PP is smooth and flat without obvious plastic deformation, which indicates a typical brittle fracture. In Figure 10b, the fracture surface is uneven after the addition of GF, and GF has a certain degree of orientation in the matrix. GF was well coated by the matrix PP, and a large number of GF fracture and pull out, mainly caused by the axial dispersion of stress along the GF.

In Figure 10c–g, for β-GFRP samples, GF was evenly dispersed in the sample and has a certain degree of orientation perpendicular to the direction of the section, and the pulled hole of GF was larger than that of GFRP sample. This indicates that the matrix of the sample became softer and more favorable to absorb more energy after the addition of β-nucleating agent. Figure 10d shows that the length of GF extracted from the sample of β-GFRP-10 is the longest, indicating that the energy absorbed by the matrix under impact stress is the largest, which is consistent with the trend of impact strength in Figure 9.

## 4. Conclusions

GFRP composites with different glass fiber content were prepared by melting blending. The mechanical properties showed that the addition of GF could improve the strength of GFRP composites, but at the same time its toughness decreased. The mechanical properties of GFRP-30 reached the maximum. The melting temperature of GFRP composite is almost constant, with only one melting peak for α-crystal PP. The effects of different contents and kinds of compatibilizers on the mechanical properties, crystallization and melting characteristics and microstructure of GFRP composites were studied. PP-g-MAH and POE-g-MAH can improve the impact strength and toughness of GFRP.

β-GFRP with different contents of nucleating agent was prepared. DSC and WAXD results show that the relative content of β-crystals increased with the increase of β-nucleating agent, and reached a maximum value of 92.8% when the content of β-nucleating agent was 0.10%. The results of mechanical properties showed that the toughness of the sample was improved, and the impact strength of the sample was improved. The tensile strength of the sample was significantly increased by the combined action of GF skeleton and β-crystal. When the addition amount of β-nucleating agent was 0.05%, the tensile strength of the sample was the highest, 43.8 MPa, and the relative increase of pure PP was 76.6%.

## Figures and Tables

**Table 1:** Crystallization and melting characteristics of GFRP with different ratio of glass fiber (GF). PP: Polypropylene.

Samples	T_c_ (°C)	ΔH_c_ (J/g)	T_m_ (°C)	ΔH_m_ (J/g)	X_α_ (%)
PP	115.0	88.7	164.9	73.2	41.4
GFRP-10	115.5	85.7	165.6	80.9	45.7
GFRP-15	116.3	78.7	164.7	73.7	41.6
GFRP-20	116.7	70.3	165.5	68.0	38.4
GFRP-25	116.7	69.7	165.1	66.8	37.7
GFRP-30	116.9	59.5	164.7	58.2	32.9

**Table 2:** Crystallization and melting characteristics of GFRP with different compatibilizers.

Samples	T_c_ (°C)	ΔH_c_ (J/g)	T_m_ (°C)	ΔH_m_ (J/g)	X_α_ (%)
PP	115.0	88.7	164.9	73.2	41.4
GFRP-10	116.9	59.5	164.7	58.2	32.9
GFRP-PP-5	118.7	68.2	164.2	66.9	37.8
GFRP-PP-10	117.9	67.4	164.2	63.8	36.0
GFRP-PP-15	117.4	65.9	164.4	62.8	35.5
GFRP-POE-5	114.7	62.1	164.8	61.0	34.5
GFRP-POE-10	114.2	54.8	163.4	52.8	29.8
GFRP-POE-15	114.3	55.2	163.4	53.4	30.2

**Table 3:** Crystallization and melting characteristics of β-GFRP composites.

Samples	T_c_ (^°^C)	T_m_^α^ (^°^C)	ΔH_α_ (J/g)	T_m_^β^ (^°^C)	ΔH_β_ (J/g)	X_α_ (%)	X_β_ (%)	Φ_β_ (%)	K_β_
PP	115.0	164.9	73.2	—	—	41.4	—	—	—
GFRP	117.2	166.2	64.3	—	—	51.9	—	—	—
β-GFRP-5	120.2	164.4	13.2	151.6	26.1	10.7	22.1	67.5	85.0
β-GFRP-10	126.7	167.0	4.1	154.4	50.4	3.3	42.7	92.8	87.2
β-GFRP-15	125.8	166.9	5.0	154.2	35.2	4.0	29.8	88.1	78.6
β-GFRP-20	126.1	166.6	7.1	153.8	46.0	5.7	39.0	87.2	79.0
β-GFRP-25	126.3	166.6	4.6	154.2	44.9	3.7	38.1	91.1	80.2
